# Ginsenoside Rg1 promotes osteogenic differentiation of rBMSCs and healing of rat tibial fractures through regulation of GR-dependent BMP-2/SMAD signaling

**DOI:** 10.1038/srep25282

**Published:** 2016-05-04

**Authors:** Yanqing Gu, Jinchun Zhou, Qin Wang, Weimin Fan, Guoyong Yin

**Affiliations:** 1 Department of Orthopedics, The First Affiliated Hospital of Nanjing Medical University, 300 Guang Zhou Road, Nanjing 210000, China

## Abstract

Fracture healing is closely related to the number and activity of bone marrow mesenchymal stem cells (BMSCs) near the fracture site. The present study was to investigate the effect of Rg1 on osteogenic differentiation of cultured BMSCs and related mechanisms and on the fracture healing in a fracture model. *In vitro* experiments showed that Rg1 promoted the proliferation and osteogenic differentiation of BMSCs. Western blot analyses demonstrated that Rg1 promoted osteogenic differentiation of BMSCs through the glucocorticoid receptor (GR)-dependent BMP-2/Smad signaling pathway. *In vivo*, X-ray examination showed that callus growth in rats treated with Rg1 was substantially faster than that in control rats after fracture. The results of H&E and Safranin-O/Fast Green staining revealed that, compared with controls, rats in the Rg1 treatment group had a significantly higher proportion of trabecular bone but a much lower proportion of fibers and cartilage components inside the callus. Micro-CT suggested that bone mineral density (BMD), percent bone volume (BV/TV), trabecular number (Tb.N), and trabecular thickness (Tb.Th) were significantly increased in the treatment group, whereas trabecular separation (Tb.Sp) was significantly reduced. Thus, Rg1 promotes osteogenic differentiation by activating the GR/BMP-2 signaling pathway, enhances bone calcification, and ultimately accelerates the fracture healing in rats.

Bone fracture healing is a remarkably complex physiological process. Delayed or poor fracture healing poses a serious threat to the life quality of patients. Fracture healing involves a series of important processes, including the proliferation and differentiation of osteoblasts and osteoclasts. Osteoblasts are crucial functional cells derived from bone marrow mesenchymal stem cells (BMSCs) and responsible for the metabolism and fracture healing of adult bones. Previous studies have suggested that osteogenic differentiation of BMSCs is regulated by a variety of hormones and local factors. Therefore, stimulation of osteogenic differentiation of BMSCs has been considered to be an important mechanism promoting fracture healing^1^,^2^,^3^.

In addition to traditional surgical intervention, the discovery of therapeutic drugs to promote bone fracture healing without surgery has recently become a hot-botton research topic. Currently, research in China is primarily focused on herbal medicines, which are primarily complex mixtures extracted from medicinal plants and have imprecise efficacy. Rg1 is a natural medicinal extract that has a wide range of pharmacological activities, including anti-inflammatory, anti-stress and immune regulation. More importantly, it has been shown that Rg1 is a functional ligand of the glucocorticoid receptor (GR) and thus exerts its biological effects through GR activation. Physiological concentrations of dexamethasone are known to effectively induce BMSC differentiation into osteoblasts *in vitro*^4^,^5^,^6^. Previous studies have found that Rg1 can promote the proliferation and osteogenic differentiation of cultured dental pulp stem cells^7^. Moreover, a complex herbal medical film containing Rg1 has been shown to promote the healing of tibial fractures in the rabbit^8^. In the present study, we demonstrate the capability of Rg1 to promote fracture healing by inducing osteoblastic differentiation of BMSCs both *in vivo* and *in vitro* and reveal the underlying mechanism.

The BMP-2/Smad signaling pathway has been shown to play an important role in bone formation and is required for osteogenic differentiation of BMSCs as well as the synthesis and secretion of extracellular matrix by osteoblasts. BMP-2 is a vital extracellular signaling molecule that promotes bone formation by inducing osteogenic differentiation of BMSCs. Previous studies have indicated that the dexamethasone-induced osteogenic differentiation of BMSCs is mediated through the BMP-2 signaling pathway^9^,^10^,^11^. A marked increase in BMP-2 expression was also observed during Rg1-induced osteogenic differentiation of dental pulp stem cells^7^. However, no studies have been reported concerning the role of the BMP-2 signaling pathway in Rg1-induced osteogenic differentiation of BMSCs.

In the present study, we show that Rg1 stimulates the expression of BMP-2 by regulating translocation of the GR receptor into the nucleus, thus promoting osteogenic differentiation of BMSCs. We also confirm the promotional effect of Rg1 on fracture healing in a rat tibia fracture model.

## Materials and Methods

### Reagents

[Table t1]Rg1 was purchased from Shanghai Oriental Pharmaceutical Co. Ltd. (Shanghai, China). Monoclonal antibodies against p-Smad1/5, p-Smad2/3, Smad1/5, Smad2/3 and osteocalcin (OCN) were purchased from Cell Signaling Technology (Danvers, MA, USA), and monoclonal antibodies against GR, Runx2, ALP, OCN, BMP-2 and collagen I (COL1) were purchased from Abcam (Cambridge, UK). Rat noggin was purchased from R&D Systems Inc. (Minneapolis, MN, USA). The CCK8 assay kit was purchased from Dojindo Laboratories (Kumamoto, Japan) and the PCR kit from Takara Biotechnology (Shiga, Japan). The primer sequences, which were synthesized by Invitrogen Corporation (Shanghai, China), are listed in Table 2. DMEM low- and high-glucose media and fetal bovine serum were purchased from HyClone (Logan, UT, USA). Penicillin-streptomycin double-resistance was purchased from Gibco (Grand Island, NY, USA). Alizarin red, cetylpyridinium chloride (CPC), RU486 and dexamethasone were obtained from Sigma (St Louis, MO, USA). Phosphate-buffered saline (PBS), the total protein extraction kit, the cytoplasmic and membrane protein extraction kit, RIPA and PMSF were purchased from Beyotime Biotechnology (Nantong, China). The mRNA extraction kit was purchased from Qiagen (Venlo, Netherlands). The ALP staining kit was provided by Nanjing Jiancheng Bioengineering Institute (Nanjing, China).

### Culture of BMSCs

Rats were sacrificed and immediately immersed in 75% alcohol for 10 min. Bilateral femurs were removed under sterile conditions. The marrow cavity was gently and repeatedly rinsed with serum-free low glucose DMEM (L-DMEM) to obtain a single cell suspension. Cells were collected by centrifugation at 1,200 rpm for 5 min, resuspended, and cultured in flasks at 37 °C in a 5% CO2 incubator. The medium was discarded after 24-h primary culture and then changed once every 3 days. Cells at 75% to 80% confluence were digested with a 0.25% trypsin solution and observed under an inverted microscope. The digestion was terminated by addition of L-DMEM containing 10% serum when cells became round and detached. A single cell suspension was prepared and subcultured at a ratio of 1:2. The medium was changed several times to obtain pure BMSCs. The 3rd generation of BMSCs was inoculated into 6-well plates and culture plates with slides at 5 × 10^6^/mL for subsequent osteogenic differentiation. Induction medium was added when cells reached 80% confluence.

### Detection of cell proliferation

BMSCs were inoculated at 1 × 10^5^/mL in 96-well culture plates (approximately 1,000 cells in 100 μL of medium per well), 100 μL of serum-free medium was then added, and cells were starved for 6 h. The medium was replaced with complete medium containing different concentrations of Rg1 (0, 0.1, 1 and 10 μg/mL) or 10^−8^ M dexamethasone. The complete medium was replaced with 100 μL of serum-free medium containing 10% CCK8 dye after 1, 2, 3 and 4 days, and cells were cultured for an additional 1 h, at the end of which color was measured at 450 nm using a microplate reader to determine the level of proliferation in each well. Cell growth curves were generated accordingly.

According to the results of CCK-8 assay, we also investigated the newly synthesized DNA of replicating cells on day 4 using a combination of Hoechst 33342 staining and a Cell-Light EdU DNA cell proliferation kit according to the manufacturer’s instructions (RiboBio, Guangzhou, China). EdU-labeled cells were counted manually in five fields of view randomly selected from each well, and percentages were calculated.

### Detection of apoptosis by Annexin V-FITC/PI double staining

BMSCs were inoculated at 1 × 10^5^/mL in 6-cm petri dishes (approximately 30,000 cells in 3 mL of medium per well) and cultured at 37 °C in a 5% CO_2_ incubator for 24 h. The medium was replaced with complete medium containing 0, 0.1, 1 and 10 μg/mL Rg1 or 10^−8^ M dexamethasone once every 2 days. After 4 days of culture, cells were detached using trypsin, collected, washed twice with cold PBS, and resuspended in 100 μL of Annexin V binding buffer. A total 5 μL of Annexin V-FITC solution and 10 μL of PI staining solution were added, and the mixture was incubated at room temperature in the dark for 15 min. At the end of the incubation, 400 μL of Annexin V binding buffer was added, and cell apoptosis was detected by flow cytometry. The experiment was repeated three times, and results were analyzed using Cellquest Pro software. The left lower (LL) quadrant represents normal viable cells with low staining intensity of both Annexin V and PI, whereas the upper left (UL) quadrant represents Annexin V-FITC-stained (early apoptotic) cells. The lower right (LR) quadrant represents late apoptotic or necrotic cells that were strongly positive for both Annexin V and PI staining.

### Effect of Rg1 on osteogenic differentiation of cultured BMSCs

The cells were planted in 24-well plates and cultured in DMEM containing 10% FBS and 1% penicillin-streptomycin for 24 h. Wells were divided into 3 groups: dexamethasone induction, Rg1 induction, and the control group. Morphological changes of the cells were observed under an inverted microscope. After induction, cells were subjected to ALP staining using Gomori’s calcium-cobalt method. Gray-black particles or massive precipitation were observed in the cytoplasm of osteoblasts. ALP activity was evaluated by a ρ-nitrophenyl phosphate (pNPP) assay. Briefly, cells were inoculated at 1 × 10^4^/mL into 6-well plates. The medium was discarded at day 7 or 14. Cells were permeabilized in 0.1% vol/vol Triton X-100 and centrifuged. The supernatant was examined by the Nakanol method following the manufacturer’s instructions to determine the ALP activity of BMSC-derived osteoblasts. The OD value of each well was measured at 405 nm. The protein concentration in each sample was determined by the BCA method, and the ALP activity of each sample was expressed as the ratio of its OD value to its protein content.

To measure calcification in culture, cells were inoculated at 1 × 10^4^/mL in 6-well plates and stained with alizarin red as described previously. Briefly, cells were washed twice with PBS, fixed in 70% ethanol for 60 min, and incubated in 40 mmol/L alizarin red S (pH 4.2) for 10 min. The cells were then washed 3 times with PBS and observed under an inverted microscope. For quantitative measurement, 10% CPC was added to the wells for 15 min, and the amount of dye released from the extracellular matrix was quantified by measuring the OD at 562 nm.

### Real-time quantitative RT-PCR

BMSCs were treated with Rg1 or Dex with and without RU486 for 2 h, 7 days or 14 days. After treatments, adherent cells were collected, and mRNA was extracted using an mRNA extraction kit from Qiagen. The concentration and purity of mRNA were measured using a spectrometer. The reverse transcription reaction was prepared according to the manufacturer’s instructions in the reverse transcription kit (Takara), and total RNA was reverse-transcribed using a SuperScript first-strand synthesis system (Invitrogen). The specific primers specific for the genes were listed in Table 2. cDNA was amplified and quantified with a dual hybridization probe system (Roche, Indianapolis, IN, USA) in the reaction mixture (Takara). The following PCR program was used: initial denaturation at 95 °C for 2 min, 40 cycles of denaturation at 95 °C for 1 min, annealing at 55 °C for 15 s, and extension at 60 °C for 1 min.

### Knockdown of BMP-2 and Smad1 with small hairpin RNA (shRNA) interfering

Small interfering RNA targeting rat BMP-2 (shBMP2) was purchased from Santa Cruz Biotechnology (Santa Cruz, CA, USA). BMSCs (60–70% confluence) were transfected with shBMP-2 or control shRNA (shcon) according to the manufacturer’s recommendations. For shSmad1, the Lenti-shRNA vector system (pGCSIL-GFP) was constructed, packed, and purified by GeneChem (Shanghai, China), and was manipulated according to the protocol provided by the manufacturer. Three different sequences were obtained from Shanghai GeneChem, cloned and tested for silencing efficiency. The targeting sequence for shSmad1 is CCAAGAGTTCGCTCAGCTA. A pGCSIL-GFP-lentiviral vector was used as a control. Three days after transfection, western blot was performed at indicated time.

### Western blot analysis

BMSCs were inoculated at 1 × 10^5^/mL in 6-cm petri dishes (approximately 30,000 cells in 3 mL of medium per well) and cultured in different groups as described above at 37 °C in a 5% CO_2_ incubator for the designated time period. In order to prove the effect of Rg1 on the GR-dependent BMP/Smad signaling pathway, wells were divided into some groups: dexamethasone induction, Rg1 induction, Rg1 and Noggin induction, Rg1 and RU486 induction, Rg1 and sh-Smad1 induction, and the control group. The BMP-2 antagonist Noggin and GR antagonist RU486 were added to cultures 1 h before dexamethasone and Rg1 treatments. ShRNA BMP-2 and ShRNA Smad1 was added to cultures 3 days before Rg1 treatments. For the extraction of total protein, cells were washed 3 times with pre-chilled PBS and lysed in 100 μL of cell lysis buffer at 4 °C for 15 min. Cell lysates were transferred to 1.5 mL Eppendorf tubes and centrifuged at 12,000 rpm/min for 10 min. The supernatants were transferred to new Eppendorf tubes, and protein content was quantified by the BCA method or stored at −80 °C. The expression level of ALP, BMP2, Runx2, OCN, COL1, p-Smad1/5, Smad1, GR and GAPDH at each designated time point was analyzed. Briefly, equal amounts of total protein (20 μg) were separated by SDS-PAGE and transferred to a polyvinylidene difluoride membrane. The membrane was blocked in TBS buffer containing 5% skim milk and 0.1% Tween 20 at room temperature for 1 h and incubated with the appropriate primary antibody overnight at pH 7.6 at 4 °C with gentle shaking. After washing, peroxidase-labeled secondary antibodies were added, and the membranes were incubated at 37 °C for 1 h. Band intensity was detected by a Molecular Imager® ChemiDoc XRS using a chemiluminescent detection system (Bio-Rad Laboratories Inc., Hercules, CA, USA). The gray value of bands was analyzed by Image Lab 2.0 software (Bio-Rad Laboratories Inc.).

### Immunocytochemical analysis

After treatments, cells were rinsed with ice-cold PBS buffer solution and immediatelyfixed with 100% methanol (−20 °C) for 15 min. Fixed cells were washed with PBS, blocked with 10% normal fetal serum for 1 h, and then incubated at 4 °C overnight with primary antibodies. After that, cells were washed three times in PBS solution for 5 min each. Then cells were incubated with fluorescent secondary antibodies (1:400). Then, the cultures were incubated in 2 mg/mL DAPI solution inPBS for 1 min to label the nuclei and immunoreactivity was visualized by microscopic examination carried out using a Leica inverted microscope equipped for fluorescence analysis (Leica Microsystems, Wetzlar, Germany). Cells were considered to be apoptotic when they showed either fragmented or condensed (pycnotic) nuclei.

### Pharmacokinetics of Rg1 after intraperitoneal injection

Administration of Rg1 and sample collection: healthy rats were deprived of food for 6 h before the experiment but were allowed free access to water thereafter. Each rat received an intraperitoneal injection of 1.5 mL Rg1 (20 mg/kg). Approximately 1 mL of venous blood was collected from the venous plexus behind the eyes of 3 rats at each time point after Rg1 administration. Each rat was subjected to a total of 10 sample collections. Blood samples were mixed with heparin and centrifuged to isolate plasma, and 200 μL of each plasma sample was vortex-mixed with 1.2 mL of methanol for 3 min. The mixture was centrifuged at 13,000 rpm/min for 10 min. The supernatant was transferred into a new tube, mixed with 100 μL of 50 ng/mL icariin, and dried under a stream of nitrogen. The residue was dissolved in 100 μL of mobile phase by vortex mixing for 3 min. The mixture was centrifuged at 13,000 rpm/min for 10 min, and the supernatant was subjected to subsequent liquid chromatographic analyses under the following conditions: mobile phase: acetonitrile:acetic acid (10 mmol/L) = 30:70; flow rate: 0.25 mL/min; and column temperature: 30 °C. The supernatant was also analyzed by electrospray ionization mass spectrometry (ESI-MS) under the following conditions: ionization modes: positive ion mode; acquisition mode: multiple reaction monitoring (MRM); ion pairs: ginsenoside Rg1, m/z 859.5 → 637.4 and icariin, m/z 735.3 → 513.1; drying gas flow rate: 9 L/min; drying temperature: 340 °C; atomizing air pressure: 35 psi; and capillary voltage: 4,000 V.

### Creation and analyses of the rat tibial fracture model

A rat tibial fracture model was created as follows: each rat was anesthetized by intraperitoneal injection of a mixture of ketamine and xylazine. Skin tissue around the knee was disinfected. A 25G needle was inserted into the medullary cavity of the tibia through the patella tendon. A fracture was created in the middle section of the tibia by the three-point bending method using an Einhorn apparatus and confirmed by x-ray examination. Rats were then injected subcutaneously with 0.5 mg/kg Buprenorphine (Abbott labs, North Chicago, IL, USA) for three days following the fracture. The experimental group (n = 36) was treated with Rg1 at a dose of 20 mg/kg per day and control group received vehicle solution (n = 36, normal saline).On days 7, 14, 21 postoperatively,twelve rats from each group were killed and the sample harvested in preparation for molecular and histological studies. Bone samples were collected at 1, 2 and 3 weeks after fracture and examined using a Faxitron X-ray system (Wheeling, IL, USA) to evaluate fracture healing. Samples were also examined with a SkyScan 1176 Micro-CT scanner (Bruker, Kontich, Belgium) with a resolution of 24 μm and analyzed using the accompanying supporting analysis and 3-dimentional imaging software to assess callus formation. All experiments were conducted in compliance with the guidelines for the care and use of laboratory animals and approved by the Institutional Animal Care and Use Committee of Nanjing Medical University (Permit Number: IACUC-1401002).

### HE, Safranin-O/Fast Green and immunohistochemical staining

Bone callus was fixed in 10% formaldehyde solution for 12–24 h, demineralized in 5% ethylenediamine tetraacetic acid (EDTA-2Na) for 3 months, dehydrated through a graded ethanol series, cleared in xylene, embedded in paraffin, and sliced into 5-μm-thick sections. Sections were subjected to H&E and Safranin-O/Fast Green staining to evaluate the morphological changes in osteoblasts and trabecular bone and to determine the content of cartilage and collagen matrix within the callus. Sections were also incubated with the corresponding primary antibody and stained by the standard SABC method. Color development was performed using a DAB kit following the manufacturer’s instructions. Each section was observed under a light microscope, and 4 visual fields were randomly selected for measurement of the rate of positive cells and the gray value, which were measured at the same light intensity.

### Statistical analysis

All experiments were independently repeated 3 times, and data are presented as the means ± standard deviation. Analysis was performed using SPSS13.0 software. Differences between groups were compared by t-tests or one-way analyses of variance (ANOVA). *P* values smaller than 0.05 were considered to be statistically significant.

## Results

### *In vitro* experiments

#### Morphological characteristics of rat BMSCs in primary culture and subculture

After 24 h in culture, round translucent primary rat BMSCs became partially adherent. The culture medium was changed every 3 d to constantly remove nonadherent cells, and visible colonies of short spindle-shaped monocytes with a clear nucleolus were observed after 3–4 days. After 6–12 d, the adherent cells expanded and formed colonies of relatively homogeneous long spindle-shaped cells that gradually fused into each other. These cells exhibited greater homogeneity compared with primary cells. Rat BMSCs exhibited monolayer growth and contact inhibition during both primary culture and subculture.

#### Effect of Rg1 on the growth curve of rat BMSCs

**Figure 1:**
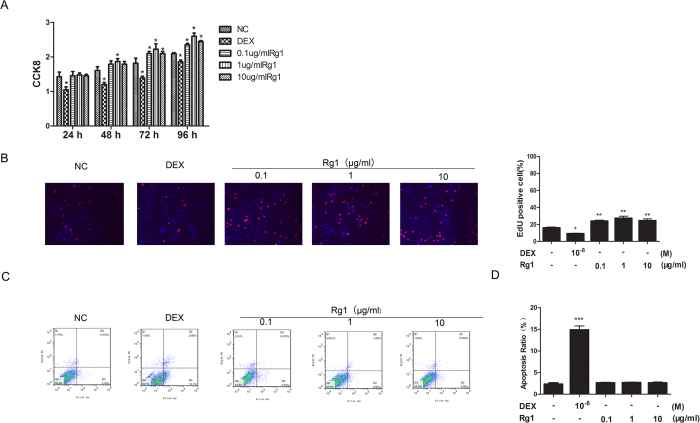
Effect of Rg1 on cell viability and apoptosis. (**A**) A CCK8 assay was performed after incubation of BMSCs (2 × 10^4^ cells/mL) with Rg1 (0.1, 1 and 10 μg/mL) or dexamethasone for 24, 48, 72 and 96 h in 96-well plates. (**B**) EdU staining of Rg1 and dexamethasone-treated BMSCs at day 4. EdU histogram. (**C**) BMSCs (1.0 × 10^5^ cells/mL) were stimulated with or without Rg1 (0.1, 1 and 10 μg/mL) or dexamethasone for 96 h in a 6-cm culture plate. Cells were collected and stained with Annexin V and propidium iodide (PI) and then examined by flow cytometry. (**D**) FACS histogram. Values are expressed as the means ± S.D. of triplicate experiments. All data are representative of three independent experiments.

The growth trends of BMSCs in all treatment groups were similar. The various concentrations of Rg1 induced BMSC proliferation, with the highest stimulation in the 1 μg/mL Rg1 group (Fig. 1A). In contrast, dexamethasone inhibited the proliferation of BMSCs. EdU labeling was performed on day 4. As shown in Fig. 1B, In the Rg1 group, more irradiated cells showed red fluorescence indicating EdU labeling. Among the dexamethasone cultures, there were fewer EdU-labeled cells (Fig. 1B).

#### Effect of Rg1 on apoptosis of rat BMSCs

Because Annexin V-FITC can bind to phospholipids in the cell membrane, while PI can pass through the incomplete cell membrane of apoptotic cells and bind to the nucleus, the Annexin V-FITC/PI method is widely used to differentiate viable and apoptotic cells. Using this method, green fluorescence reveals the cell membrane of viable cells under a fluorescence microscope, whereas red fluorescence highlights the nuclei of apoptotic cells (Fig. 1C). As shown in Fig. 1C, the proportion of viable cells in all Rg1-treated groups was greater than 95%, and there was no significant difference in cell viability among these groups (*P* > 0.05). The apoptosis rate in the dexamethasone group was approximately 10%, which was significantly higher than in the other groups (Fig. 1C,D).

#### Effect of Rg1 and dexamethasone on osteogenic differentiation of BMSCs

**Figure 2:**
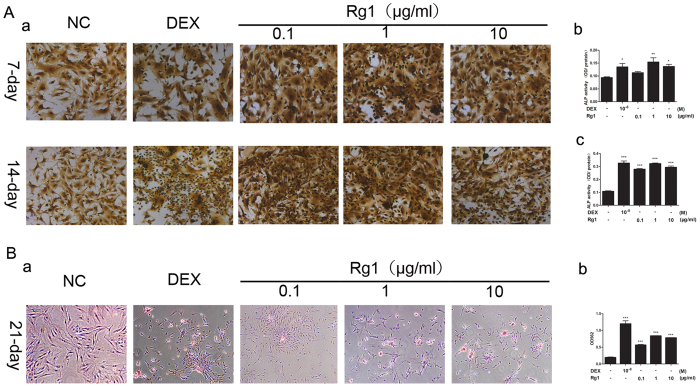
Stimulatory effect of Rg1 on osteogenic differentiation of BMSCs. (**A**) ALP staining of Rg1 and dexamethasone-treated BMSCs at days 7 and 14. (**B**) Alizarin red staining of Rg1- and dexamethasone-treated BMSCs at day 18 for the comparison of mineralization in the different groups. Values are expressed as means ± SD of triplicate experiments.*P < 0.05, **P < 0.01, ***P < 0.001.

**Figure 3:**
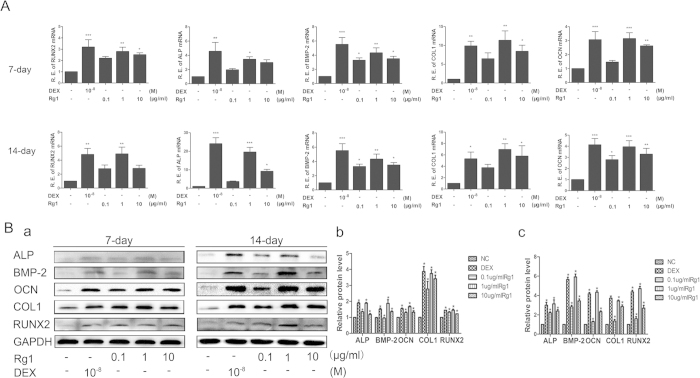
Effect of Rg1 on the expression of genes related to osteogenic differentiation of BMSCs. (**A**) RT-PCR analysis of mRNA expression of osteogenic genes, including BMP-2, Runx2, OCN, COL1 and ALP, in BMSCs treated with Rg1 or dexamethasone for 7 or 14 days. (**B**) Western blot analysis of the expression of osteogenic proteins, including BMP-2, Runx2, OCN, COL1 and ALP, in BMSCs treated with Rg1 or dexamethasone for 7 or 14 days. Values are expressed as means ± SD of triplicate experiments.*P < 0.05, **P < 0.01, ***P < 0.001.

The expression of ALP in both Rg1- and dexamethasone-treated groups was significantly higher than in the controls at 7 and 14 days after induction, suggesting that Rg1 stimulated the expression of ALP (Fig. 2A). Moreover, the number of calcified nodules and the content of calcium in all Rg1 groups were significantly higher compared with the dexamethasone group. This suggests a stimulatory effect of Rg1 on both ALP expression and calcification of BMSCs that was comparable to that of dexamethasone. RT-PCR and Western blot analysis also showed that both Rg1 and dexamethasone increased the expression of genes related to osteogenic differentiation, including ALP, OCN, COL1, BMP-2, Runx2 (Fig. 3A,B).

#### Effect of Rg1 on the GR-dependent BMP/Smad signaling pathway

**Figure 4:**
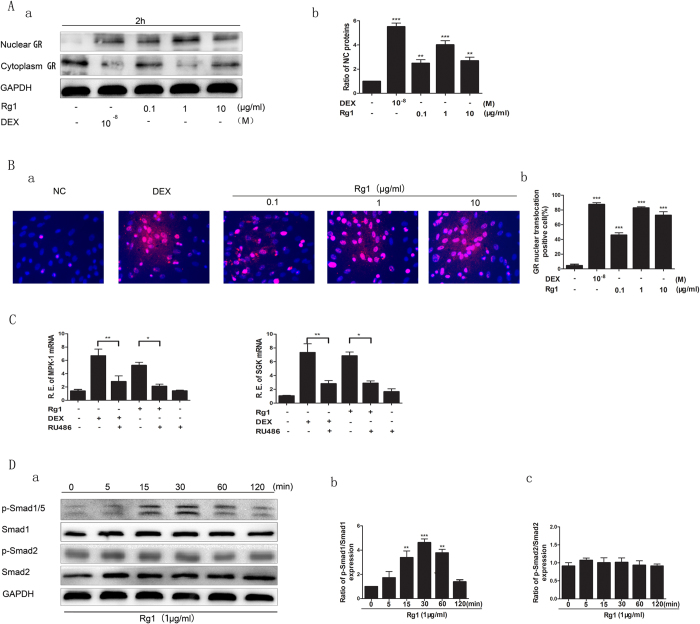
Effect of Rg1 on the GR-dependent BMP/Smad signaling pathway. (**A**) Western blot analysis of the effect of Rg1 and dexamethasone on translocation of the GR from the cytoplasm into the nucleus, (**B**) Immunofluorescence imaging demonstrated that Rg1 and dexamethasone promoted GR nuclear translocation. (**C**) C, The transcription of the responsive genes were analyzed by semi-quantitative RT-PCR. 1 μg/ml Rg1 mimicked Dex to induce the transcription of glucocorticoid-responsive genes such as MKP-1 and SGK, while RU486 could abolish the effects. (**D**) Western blot analysis of the effect of Rg1 and dexamethasone on the phosphorylation level of Smad1/5 and Smad2. Values are expressed as means ± SD of triplicate experiments. *P < 0.05, **P < 0.01, ***P < 0.001.

**Figure 5:**
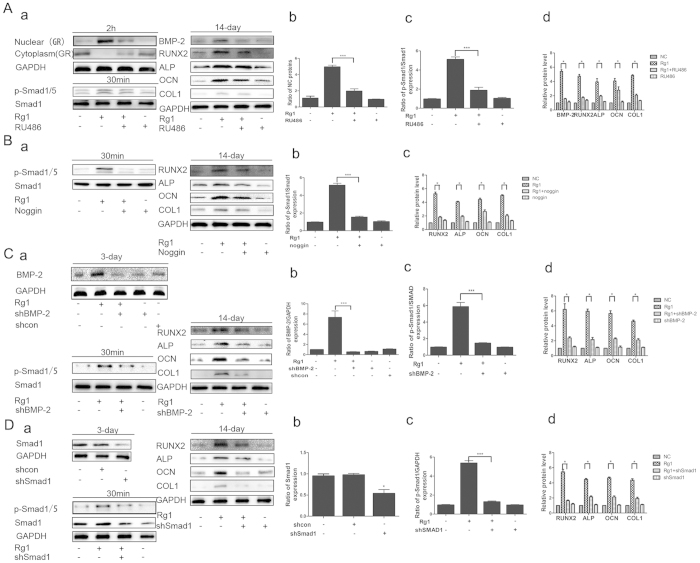
Effect of Rg1 on osteogenic differentiation of BMSCs after blocking translocation of the GR into the nucleus inhibiting the effect of BMP-2, transfection with shBMP-2 or transfection with shSmad1. Western blot analysis of the effect of Rg1 on expression of the osteogenic proteins BMP-2, RUNX2, ALP, OCN and COL1 in cells treated with (**A**) the GR antagonist RU486 (**B**) the BMP-2 inhibitor noggin (**C**) shBMP-2 or (**C**) shSmad1. Values are expressed as means ± SD of triplicate experiments. *P < 0.05, **P < 0.01, ***P < 0.001.

The GR is a ligand-dependent transcription factor that is activated upon binding by glucocorticoid (GC). The activated GR forms a homodimer that is translocated into the cell nucleus, where it regulates gene transcription. Rg1 is a plant-derived glucocorticoid receptor agonist. Western blot analysis confirmed that the GR translocated from the cytoplasm to the nucleus in both Rg1 and dexamethasone groups 2 h after treatment (Fig. 4A). Consistent with the western blot of cytoplasmic and nuclear fractions, results from immunofluorescence assay further demonstrated GR staining of Rg1-treated neurons in nuclei were much intense than that of control (Fig. 4B). In addition, we assessed the effect of Rg1 on induction of SGK and MKP-1, two well-known glucocorticoid-responsive genes. Rg1 mimicked Dex to induce the transcription of glucocorticoid-responsive genes such as mitogen-ativated protein kinase phosphatase-1 (MKP-1) and glucocorticoidregulated kinase (SGK), while RU486 could abolish the effects, these data indicated that Rg1 exerted some effects on GR activation (Fig. 4C). We also evaluated the effect of the specific GR antagonist RU486 on the induction of osteogenic differentiation by Rg1 to validate whether Rg1-induced osteogenic differentiation was mediated by the GR. Our results showed that RU486 blocked the Rg1-induced increase in p-Smad1/5 levels and significantly downregulated the expression of osteogenic proteins, including ALP, OCN, COL1, BMP-2 and Runx2, after 14 days of treatment, indicating the regulatory effect of GR on the expression of BMP-2 (Fig. 5A).

Furthermore, the p-Smad1/5 level at 0, 5, 15, 30, 60 and 120 min after treatment was compared between all groups. As shown in Fig. 4D, the expression of p-Smad1/5 in the Rg1 group started to increase 5 min after the addition of Rg1, although it was not significantly higher than in the control group. However, p-Smad1/5 levels in the Rg1 group continued to increase and peaked at 30 min, by which time they were significantly higher than in the control group (*P* < 0.05). By 2 h, p-Smad1/5 expression in the Rg1 group had returned to near control levels. We further examined the effect of noggin, a BMP-2 antagonist, on Rg1-stimulated cells. Our results demonstrated that the expression of p-Smad1/5/8 in the Rg1 group was significantly elevated compared with the control group after 1 h of treatment (*P* < 0.01), whereas pretreatment with 100 nmol noggin and shBMP-2 blocked the stimulatory effect of Rg1 on p-Smad1/5/8 expression. This suggested that BMP-2 is located upstream of Smad1/5 and therefore mediates the Rg1-induced activation of Smad1/5/8 in BMSCs (Fig. 5B,C). Additionally, there was no effect of Rg1 on the expression of p-Smad2. Moreover, we performed knockdown analysis using Smad1 shRNAs to confirm the role of Smad1 in the regulatory effect of Rg1-induced osteogenic protein expression. Knockdown study revealed that Smad1 activates downstream of GR-dependent BMP/Smad signaling pathway in the induction of Rg1 (Fig. 5D).

**Figure 6:**
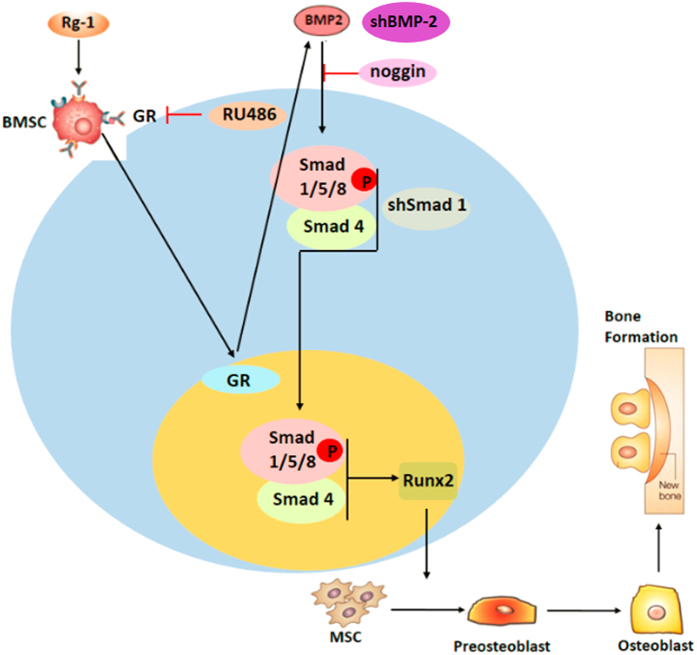
Effect of Rg1 on the GR-dependent BMP/Smad signaling pathway.

Effect of Rg1 on the GR-dependent BMP/Smad signaling pathway (Fig. 6).

### *In vivo* experiments

#### Pharmacokinetics of Rg1 in rats after intraperitoneal injection of 20 mg/kg

**Figure 7:**
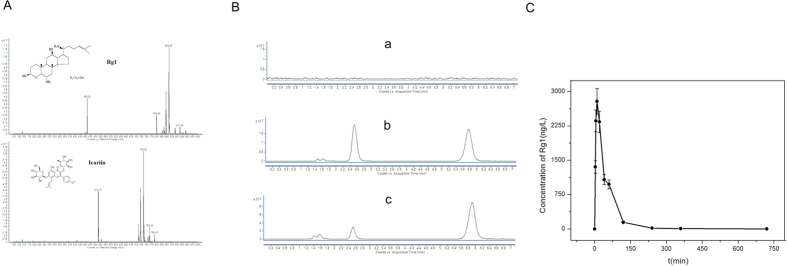
Pharmacokinetic analysis of rats administered an intraperitoneal injection of 20 mg/kg Rg1. (**A**) Full mass spectrum scan of Rg1 and icariin. (**B**) (a) Chromatogram of plasma samples of blank control rats. (b,c) HPLC chromatogram of blank control plasma samples containing Rg1 and the icariin reference and of plasma samples of rats treated with Rg1. (**C**) The pharmacokinetic curve of Rg1 showing the characteristics of Rg1 in rats at each time point.

The full scan spectra of ginsenoside Rg1 and icariin are shown in Fig. 7A. Plasma was obtained from rats in the control group and subjected to high performance liquid chromatography (HPLC) as described in section 2.8 (Fig. 7B (a)). Rg1 and the reference icariin were added to the control plasma and examined by HPLC (Fig. 7B (b)). Plasma was also obtained from rats in the treatment group and subjected to HPLC (Fig. 7B (c)). The retention times of Rg1 and icariin were 2.364 and 5.603 min respectively, indicating that endogenous substances in rat plasma do not interfere with the determination of Rg1 by HPLC.

Standard curve and linear range: 100 μL of plasma was obtained from rats in the control group, and 50 μL of various concentrations of Rg1 and icariin was added, with 3 replicates of each concentration. These solutions were analyzed by HPLC as described in section 2.8 to create the standard curve. A linear regression equation was obtained by a weighted least squares regression method, with the analyte concentration on the x-axis and the ratio of peak area of Rg1 and icariin in chromatograms on the y-axis. The standard curve of Rg1 was Y = 0.4223x + 0.0230 with a correlation coefficient of 0.996 (weight W = 1/CC) and a linear range of 1–200 ng/L, and Y = 0.5353x + 2.0685 with a correlation coefficient of 0.995 (weight W = 1/CC) and a linear range of 200–5,000 ng/L.

Results of the pharmacokinetic tests: the pharmacokinetic characteristics of Rg1 in rats at different time points are shown in Fig. 7C.

#### X-ray and Micro-CT findings

**Figure 8:**
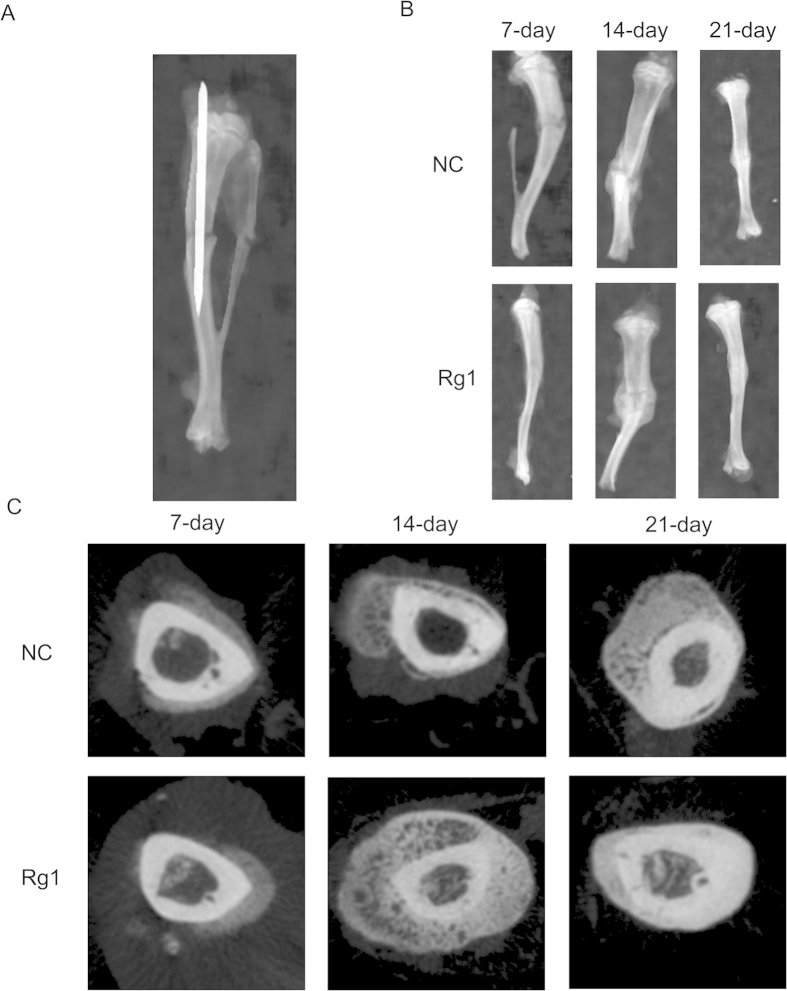
Creation of the rat fracture model. A fracture was created in the middle section of the tibia by the three-point bending method using an Einhorn apparatus. Stimulatory effect of Rg1 on healing of the tibial fracture. (**A**) X-ray examination showing the stable rat tibia fracture. (**B**) Comparison of X-ray images of rat tibial fractures between the Rg1 group and the control group at 7, 14 and 21 days after fracture. (**C**) Comparison of micro-CT images of rat tibial fractures between the Rg1 group and the control group at 7, 14 and 21 days after fracture.

Postoperative condition of the rats: no animals died during surgery, and the rat tibial fracture model was successfully created, as demonstrated by X-rays and CT. X-ray and CT examination revealed a clear fracture line in the tibia and good reduction of the fracture in both Rg1 and control groups immediately after surgery. After 1 week, the tibia and fibula fracture lines remained obvious in the control group, whereas both lines in the Rg1 group had become blurred, and the fracture was surrounded by a small amount of callus. After 2 weeks, the amount of callus in the Rg1 group was much greater than that in the control group. The blurred fracture line in the tibia remained faintly visible. After 3 weeks, while the tibial fracture line in the control group remained visible, the line in the Rg1 group had disappeared (Fig. 8). Micro CT identified a significant increase in BMD and several bone histomorphometric parameters, including BV/TV, Tb.N and Tb.Th, and a significant decrease in Tb.Sp (Table 1).

#### H&E and Safranin-O/Fast Green staining

**Figure 9:**
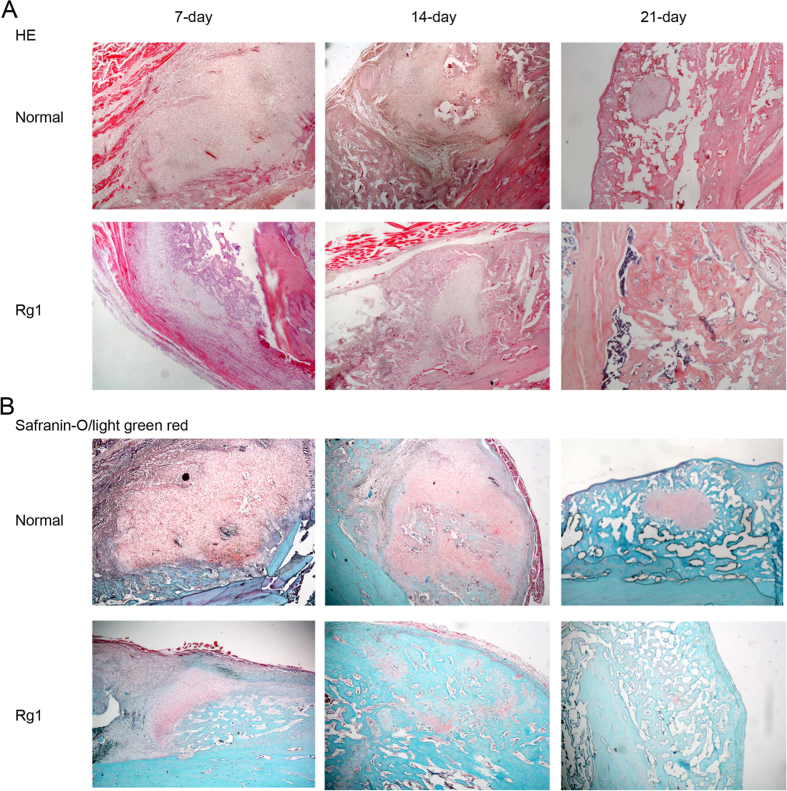
Effect of Rg1 on morphological changes in the fracture callus. (**A**) H&E staining of bone sections showing morphological changes in cancellous and trabecular bone in the Rg1 and control groups at 7, 14, and 21 days after fracture. (**B**) Safranin-O/Light Green Red staining of bone sections showing comparison of the proportion of fibrous and osseous callus within the fracture site in Rg1 and control groups at 7, 14, and 21 days after fracture.

Although sections in both Rg1 and control groups revealed a gradual transition from fibrous callus to osseous callus, there was a significant difference in the proportion of the two types of callus between the two groups. After 1 or 2 weeks, rats in the Rg1 group had significantly more osseous callus and developed mature trabecular bone, whereas the control rats had relatively more fibrous callus and a large number of cartilage-like cells. After 3 weeks, while the Rg1-treated rats had only osseous callus, fibrous callus was still present in the control group (Fig. 9A).

Sections were also stained with Safranin-O/Light Green Red to detect the fracture line and to distinguish interstitial collagen fibers, cartilage and trabecular bone within the callus. As shown in Fig. 9B, the cartilage area in the Rg1 group was significantly smaller compared with that in the control group at 7, 14 and 21 days after fracture, which was consistent with the H&E results.

## Discussion

Bone fracture healing is a unique physiologic process during which the bone repairs itself locally. The process involves both osteoblasts and osteoclasts, and the generation of a large number of osteoblasts during the early stage of facture healing is crucial. Several studies have confirmed that osteoblasts that proliferate after a fracture differentiate from mesenchymal stem cells and marrow stromal cells during the inflammatory and hematoma stages. Mature osteoblasts secrete a matrix material called osteoid and later differentiate into osteocytes after becoming embedded in the osteoid. This subsequently develops into bone matrix by calcification followed by the formation of bone tissues to complete the process of bone healing^1^,^2^,^3^.

Currently, *in vitro* induction of osteogenic differentiation of BMSCs can be achieved by culturing cells in medium containing dexamethasone, ascorbic acid, and β-glycerophosphate. As the most critical component, dexamethasone can promote ALP expression and activity in BMSCs and increase collagen synthesis in the extracellular matrix, thus promoting BMSC differentiation into osteoblasts^4^,^5^,^6^. Previous studies have revealed that Rg1 is a plant-derived glucocorticoid receptor agonist, which has a significant anti-inflammatory effect comparable to that of dexamethasone^12^,^13^. However, no studies concerning any stimulatory effect of Rg1 on osteogenic differentiation of BMSCs have been reported. In the present study, Western blot analysis showed that Rg1 stimulates translocation of the GR into the nucleus and promotes osteogenic differentiation of BMSCs, suggesting that Rg1-induced osteogenic differentiation of BMSCs might be mediated via translocation of the GR into the nucleus. Dexamethasone is known to inhibit cell proliferation and increase apoptosis during osteogenic differentiation of BMSCs^14^, while in our study, Rg1 promoted the proliferation of BMSCs without inducing apoptosis. Previous studies have found that Rg1 promotes proliferation of BMSCs by non-ligand binding to the estrogen receptor^15^. Rg1 also did not induce apoptosis in our study, which is likely attributable to its plant estrogen-like effect.

In the present study, Rg1 stimulated cellular ALP activity and significantly increased the number of calcified nodules as well as the expression of osteocalcin, COL1, BMP2 and Runx2 at 7 and 14 days after induction, suggesting that Rg1 has a stimulatory effect on osteogenic differentiation of BMSCs. Osteoblasts are the primary functional cells during bone formation; they are distributed on the bone surface and are responsible for the synthesis, secretion and mineralization of the bone matrix. Osteogenic markers include ALP, OCN, COL1, and Runx2. The homodimeric glycoprotein ALP is an important protein involved in bone metabolism and is considered to be an early indicator of the maturation of extracellular matrix. ALP activity is thought to represent the early stage of osteogenic differentiation, and cellular ALP levels gradually increase as osteogenic differentiation progresses^16^,^17^,^18^. Another osteogenic marker, type I collagen, is one of the main components of the extracellular matrix secreted by osteoblasts, and it provides a structural framework for the maturation of extracellular matrix and the development of calcified nodules^19^,^20^,^21^. Osteocalcin is an important non-collagenous component of bone matrix that is secreted only by osteoblasts, primarily during the stage of mineral formation, and it is also regarded as an osteogenic marker^22^. The number of calcified nodules can also indicate the extent of mineralization. Core binding factor al (Cbfal), also known as Runt-related gene 2 (Runx2) belongs to the Runt/Cbfa transcription factor family, members of which can directly stimulate the transcription of various genes related to osteogenic differentiation of BMSCs, including osteocalcin, osteopontin, and collagen I. The expression of Runx2 marks the beginning of osteogenic differentiation; therefore, the protein is the first and most specific marker of bone formation^23^. In our study, expression of all of these osteogenic markers was significantly increased in the Rg1 group, indicating the stimulatory effect of Rg1 on osteogenic differentiation of BMSCs. Our results are consistent with the regulatory effect of Rg1 on osteogenic differentiation of dental pulp stem cells^7^. These findings strongly suggest that Rg1 may be a potential inducer of osteogenic differentiation.

The biological effects of GC are known to be mainly mediated by the GR. Dexamethasone induces the differentiation of BMSCs into osteoblasts by increasing the expression of BMP-2^24^. In this study, we investigated whether the stimulatory effect of Rg1 on osteogenic differentiation of BMSCs was mediated by activation of the GR. We used RU486 to block the GR and found that the level of BMP-2, as well as other proteins related to osteogenic differentiation, was significantly reduced, suggesting that Rg1 promotes osteogenic differentiation by regulation of the GR. This finding also confirmed that the GR is an upstream factor in the BMP-2/Smad signaling pathway.

BMP-2 belongs to the transforming growth factor beta (TGF-β) superfamily and is an important regulator of bone formation. Previous studies have suggested that BMP-2 may accelerate BMSC differentiation into osteoblasts through the stimulation of genes related to osteogenic differentiation. BMP-2 binds to receptors on the cell membrane, resulting in the phosphorylation of intracellular Smad1, Smad5 or Smad8 followed by their translocation from the cytoplasm into the nucleus. In the nucleus, the metal ion-dependent adhesion site (SmadS) heteromeric complex binds to its specific target genes with the help of several DNA binding proteins, regulates downstream transcription factors, such as cbfa1, and further activates the expression of osteogenic markers, including ALP, OCN and COL1^9^,^10^,^11^. In the present study, we observed an increase in BMP-2 expression in the Rg1 group during osteogenic differentiation. We further demonstrated that the expression of p-Smad1/5, ALP, OCN, COL1 and Runx2 by Rg1 was reduced in the presence of the BMP-2 antagonist noggin and siBMP-2, indicating that the stimulatory effect of Rg1 on osteogenic differentiation was likely mediated by increased expression of BMP-2.

We used HPLC to further examine the pharmacokinetics of Rg1 in rats administered an intraperitoneal injection of Rg1, and we confirmed that Rg1 would be able to reach the hematoma site through blood circulation within 30 min after injection.

Several studies on the effect of Rg1 on fracture healing using experimental animal models have been published. We explored the effects of Rg1 on fracture healing from the perspective of bone histomorphometry. A closed rat tibial fracture model was created using a special modeling device that does not cause the thermal damage typical of osteotomy tools. Moreover, there was no loss of BMSCs because no flush is needed in the surgery. In the present study, fracture healing in the Rg1 group was substantially promoted at all time-points compared with the control group, as demonstrated by X-ray examination. On day 21 after treatment, the fracture line had disappeared, and the marrow cavity was restored in the Rg1 group, indicating a stimulatory effect of Rg1 on fracture healing. Moreover, the micro-CT analysis revealed increased BMD and histomorphometry parameters, including BV/TV and Tb.N values, together with a decrease in Tb.Sp value, suggesting that bone mass increased. The increased bone density and mass of the tibia provides direct evidence in support of the osteogenic effect of Rg1. Bone sections were examined by H&E staining and Safranin-O/Light Green Red staining, which revealed that Rg1 greatly accelerated the transformation of fibrous callus into osseous callus. These results suggest that Rg1 promotes the calcification of cartilage and osteogenesis during the middle and late phases of fracture healing by stimulating the proliferation and differentiation of osteoblasts, improving bone strength, and thus accelerating fracture healing.

Previous studies have shown that Rg1 can promote fracture healing by regulating angiogenesis^8^. In the present study, we demonstrate that the mechanism of Rg1-induced osteogenesis is similar to that of dexamethasone and is mediated through regulation of the GR-dependent BMP-2/Smad signaling pathway. We further demonstrate that Rg1 promotes fracture healing by significantly increasing the bone mass and density of the fracture callus.

## Additional Information

**How to cite this article**: Gu, Y. *et al.* Ginsenoside Rg1 promotes osteogenic differentiation of rBMSCs and healing of rat tibial fractures through regulation of GR-dependent BMP-2/SMAD signaling. *Sci. Rep.* **6**, 25282; doi: 10.1038/srep25282 (2016).

## Figures and Tables

**Table 1 t1:** Micro-CT assessment of rat tibial fractures between the Rg1 group and the control group at 1, 2 and 3 weeks.

	1w	1w + Rg1	2w	2w + Rg1	3w	3w + Rg1
BV/TV (%)	29.17 ± 2.281	36.28 ± 1.069*	52.36 ± 1.314	53.91 ± 2.121*	56.82 ± 2.077	64.71 ± 0.9444*
BS/TV (1/mm)	2.692 ± 0.8699	4.416 ± 0.4582	4.522 ± 0.1187	5.316 ± 0.2950*	5.294 ± 0.3111	5.792 ± 0.1764*
Tb.N (/mm)	0.753 ± 0.1557	1.082 ± 0.029*	1.404 ± 0.0546	1.643 ± 0.068*	1.825 ± 0.073	2.369 ± 0.163*
Tb.Sp (μm)	0.801 ± 0.1328	0.6556 ± 0.0534	0.633 ± 0.056	0.5136 ± 0.023*	0.5405 ± 0.04841	0.4167 ± 0.069*
Tb.Th (μm)	0.21 ± 0.014	0.3195 ± 0.041	0.349 ± 0.017	0.4478 ± 0.026*	0.5086 ± 0.017	0.6412 ± 0.04*
BMD	0.459 ± 0.03	0.726 ± 0.00853*	0.633 ± 0.0388	1.681 ± 0.123*	1.663 ± 0.09572	2.042 ± 0.03478*

**Table 2:** Sequences of primers of the genes in RT-PCR analysis.

ALP	TCACTTCCGCCCGGAACCCT	TGTCCTGCCGGCCCAAGAGA
OCN	GGCAGGTGCAAAGCCCAGCG	GGGCTGGGGCTCCAAGTCCAT
COL1	CTGCCCCTCGCAGGGGTTTG	GCCTGCACATGTGTGGCCGA
BMP-2	TGAACACAGCTGGTCTCAGG	ACCCCACATCACTGAAGTCC
RUNX2	TCACAAATCCTCCCCAAGTGG	GAATGCGCCCTAAATCACTGA
GAPDH	CCATCATGAAGTGTGACGTG	ACATCTGCTGGAAGGTGGAC
MKP-1	CTGCTTTGATCA ACGTCTCG	AAGCTGAAGTTGGGGGAGAT
SGK	TGCTCTATGGCCTG CCTCCGTTC	GTCACTGGGCCCGCTCACATTTG
